# Sustained Diurnal Stimulation of Cyclic Electron Flow in Two Tropical Tree Species *Erythrophleum guineense* and *Khaya ivorensis*

**DOI:** 10.3389/fpls.2016.01068

**Published:** 2016-07-19

**Authors:** Wei Huang, Ying-Jie Yang, Hong Hu, Kun-Fang Cao, Shi-Bao Zhang

**Affiliations:** ^1^Key Laboratory of Economic Plants and Biotechnology, Kunming Institute of Botany – Chinese Academy of Sciences, KunmingChina; ^2^Yunnan Key Laboratory for Wild Plant ResourcesKunming, China; ^3^Key Laboratory of Tropical Forest Ecology, Xishuangbanna Tropical Botanical Garden – Chinese Academy of SciencesMengla, China

**Keywords:** cyclic electron flow, photoprotection, photosystem I, photosystem II, recovery

## Abstract

The photosystem II (PSII) activity of C_3_ plants is usually inhibited at noon associated with high light but can be repaired fast in the afternoon. However, the diurnal variation of photosystem I (PSI) activity is unknown. Although, cyclic electron flow (CEF) has been documented as an important mechanism for photosynthesis, the diurnal variation of CEF in sun leaves is little known. We determined the diurnal changes in PSI and PSII activities, light energy dissipation in PSII and the P700 redox state in two tropical tree species *Erythrophleum guineense* and *Khaya ivorensis* grown in an open field. The PSI activity (as indicated by the maximum quantity of photo-oxidizable P700) was maintained stable during the daytime. CEF was strongly activated under high light at noon, accompanying with high levels of non-photochemical quenching (NPQ) and PSI oxidation ratio. In the afternoon, CEF was maintained at a relatively high level under low light, which was accompanied with low levels of NPQ and P700 oxidation ratio. These results indicated that CEF was flexibly modulated during daytime under fluctuating light conditions. Under high light at noon, CEF-dependent generation of proton gradient across the thylakoid membranes (ΔpH) mainly contributed to photoprotection for PSI and PSII. By comparison, at low light in the afternoon, the CEF-dependent formation of ΔpH may be important for PSII repair via an additional ATP synthesis.

## Introduction

Light is the driving force for photosynthesis. However, excess light excitation could lead to photoinhibition (Powles, 1984; Barber and Andersson, 1992; Aro et al., 1993). High light stress usually causes selective photoinhibition of photosystem II (PSII; Barber and Andersson, 1992; Prasil et al., 1992; Asada, 1996, 1999). Photoinhibition of PSII occurs only when the rate of photodamage to PSII exceeds the rate of its repair (Aro et al., 1993; Murata et al., 2007; Takahashi and Murata, 2008; Takahashi et al., 2009). Under conditions in which absorbed light is in excess of the requirement of photosynthesis, excess light energy leads to the production of reactive oxygen species (ROS; Murata et al., 2007). It has been indicated that the ROS accelerate PSII photoinhibition mainly through inhibition of the repair of photodamaged PSII (Nishiyama et al., 2001, 2004), although some exceptions indicate that ROS cause direct photodamage to PSII (Oguchi et al., 2009). To avoid severe irreversible photodamage to PSII, plants have several photoprotective mechanisms to diminish the rate of photodamage and favor the repair of photodamaged PSII, including adjusting PSII connectivity (Zivcak et al., 2014), thermal energy dissipation (Niyogi et al., 1998, 2001), and cyclic electron flow (CEF) around PSI (Munekage et al., 2002, 2004; Takahashi et al., 2009; Huang et al., 2011; Suorsa et al., 2012; Brestic et al., 2015; Zivcak et al., 2015).

Plants have the ability to dissipate excess light energy harmlessly as heat through non-photochemical quenching (NPQ; Niyogi et al., 1998, 2001), which is dependent on not only xanthophyll cycle, i.e., the de-epoxidation of violaxanthin to zeaxanthin via antheraxanthin (Demmig-Adams, 1990), but also the establishment of a proton gradient across the thylakoid membranes (ΔpH; Munekage et al., 2002, 2004; Nandha et al., 2007). Leaves grown under high light usually have stronger capacity to fulfill the NPQ process by the enhancements of xanthophyll cycle and CEF activities (Miyake et al., 2005; Ballottari et al., 2007). CEF-dependent generation of ΔpH helps to alleviate photoinhibition by at least two different photoprotection mechanisms: one is linked to thermal energy dissipation (qE) generation and prevents the inhibition of the repair of photodamaged PSII, and the other is independent of qE and suppresses the photodamage to PSII (Takahashi et al., 2009). As a result, impairment of CEF could lead to severe photoinhibition of PSII under high light (Takahashi et al., 2009). The increases in leaf-to-air vapor pressure deficit and air temperature at noon induce a decrease in photosynthetic rate (Zhang et al., 2009), which subsequently results in PSII photoinhibition (Takahashi and Murata, 2005, 2006; Murata et al., 2007). Meanwhile, plants display high levels of NPQ at noon. Therefore, we speculate that CEF is strongly stimulated in sun leaves at noon associated with high light to promote NPQ.

Photoinhibited PSII at noon could be repaired fast in the afternoon (Allen et al., 2000; He and Chow, 2003; Hendrickson et al., 2004a). It has been reported that photoinhibition of PSII can be quickly repaired at low light unless PSI activity is extremely inhibited (Sundby et al., 1993; He and Chow, 2003; Zhang and Scheller, 2004; Huang et al., 2010a). The fast repair of PSII photoinhibition is dependent on rapid synthesis of bioenergy (ATP) which requires the generation of ΔpH across thylakoid membranes (Allakhverdiev et al., 2005). It has been indicated that CEF can help ATP synthesis under low light in *Arabidopsis thaliana* (Nishikawa et al., 2012), rice (Yamori et al., 2011), and tobacco (Wang et al., 2006; Huang et al., 2015). A previous study indicated that CEF was stimulated under low light during the recovery after chilling-induced photoinhibition of PSII (Huang et al., 2010b). Since ATP synthesis might regulate the repair of PSII (Allakhverdiev et al., 2005), we speculate that CEF is stimulated in the afternoon to generate the ATP and then help the fast repair of PSII photoinhibition.

Photoinhibition of PSI is mainly caused by the oxidation of hydroxyl radical that is usually generated by a reaction between hydrogen peroxide and a reduced metal ion in a process called the Fenton reaction (Asada and Takahashi, 1987; Sonoike, 1996a,b). Thus, there are two necessary mechanisms responsible for PSI photoinhibition: over generation of hydrogen peroxide and over reduction of PSI reaction centers (Sonoike, 1995; Munekage et al., 2002, 2004; Tikkanen et al., 2014). Active electron flow from PSII is necessary for photoinhibition of PSI in chilled cucumber (Havaux and Davaud, 1994; Sonoike, 1995). In the PROTON GRADIENT REGULATOR5 (*pgr5*) plants of *A. thaliana*, PSI is extreme sensitive to high light stress (Munekage et al., 2002; Suorsa et al., 2012; Kono et al., 2014; Tikkanen et al., 2014). Photoinhibition of PSI severely affects CO_2_ assimilation and photoprotection in wheat leaves (Brestic et al., 2015; Zivcak et al., 2015). CEF around PSI has been documented as an important mechanism for protecting PSI from photoinhibition under high light because the activation of CEF contributes to the oxidation of P700 and alleviates over-reduction of the PSI acceptor side (Munekage et al., 2002, 2004; Kono et al., 2014; Tikkanen et al., 2014, 2015). P700^+^ can dissipate excess excitation energy harmlessly and thus alleviate photoinhibition of PSI. Moreover, stimulation of CEF could alleviate the over accumulation of the reducing power NADPH and then diminished the generation of ROS in PSI acceptor side, especially at high temperature (Wang et al., 2006; Essemine et al., 2016). High levels of light condition and leaf temperature are typical conditions in clear days in summer. We speculate that stimulation of CEF under high light at noon favors the photoprotection for PSI.

In our present study, we determined the diurnal changes in PSI and PSII activities, light energy quenching in PSII and P700 redox state for leaves of two tropical tree species *Erythrophleum guineense* and *Khaya ivorensis* grown in an open field. The following questions were addressed: (1) Is PSI activity maintained stable during the daytime? (2) Is CEF stimulated at low light in the afternoon for the fast repair of PSII photoinhibition? (3) Is the role of CEF regulated flexibly during the daytime with fluctuation of light condition?

## Materials and Methods

### Plant Materials

**FIGURE 1:**
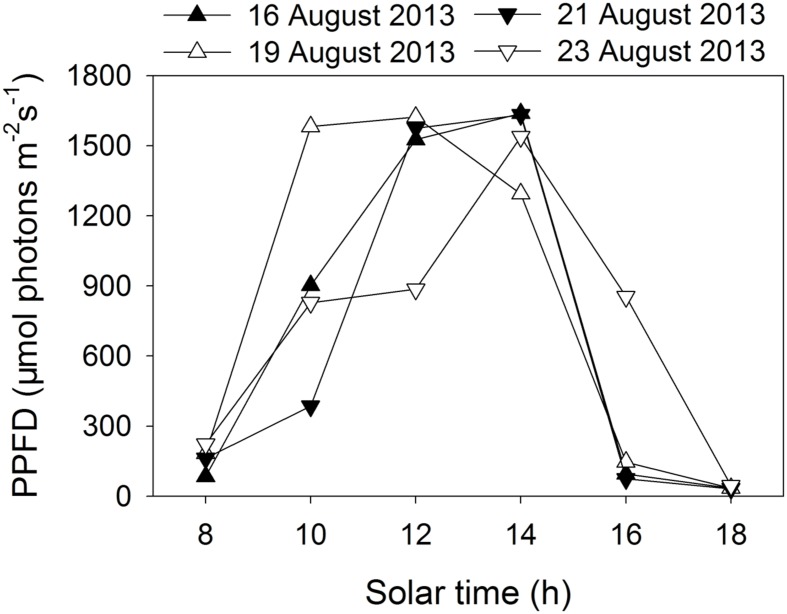
**Diurnal change in photosynthetic photon flux density (PPFD) on 4 days in summer (16 August, 19 August, 21 August, 23 August 2013) in an open site**.

Two tropical tree species were chosen for the present study. *E. guineense* G. Don (Fabaceae) is a large canopy species native to tropical Africa. *K. ivorensis* A. Chev (Meliaceae) is a large canopy species found in various habitat types in west and central tropical Africa but is most abundant in wet undisturbed evergreen forests. Potted 2-years-old seedlings of *E. guineense* and *K. ivorensis* were used for experiments. Plants of these two species grown well in an open field in Xishuangbanna Tropical Botanical Garden (21°54′ N, 101°46′ E). We conducted all measurements in 4 days in summer (16 August, 19 August, 21 August, and 23 August in 2013). The diurnal changes in photosynthetic photon flux density (PPFD) are indicated in **Figure 1** During these periods, the air temperature changed from 20°C at night to 32°C in the daytime.

### Chlorophyll Fluorescence and P700 Redox State Measurements

We synchronously measured the light responses of chlorophyll fluorescence and P700 redox state at 25°C with Dual PAM-100 (Heinz Walz, Effeltrich, Germany) connected to a computer with control software. In the present study, a 635 nm LED was used as actinic light. We conducted the measurements of light response curves in the morning. In order to eliminate the effect of photoinhibition on measurements of light response curves, the potted plants were transferred to a shade place the night before. Mature leaves were light-adapted (360 μmol photons m^-2^ s^-1^) for at least 20 min at 25°C before the measurement of light response curves, and light-adapted photosynthetic parameters were recorded after 3 min exposure to each light intensity (1957, 1599, 1292, 830, 536, 221, 131, 58, and 18 μmol photons m^-2^ s^-1^).

The diurnal PSI and PSII parameters were conducted on five to six intact leaves on clear days every 2 h in daytime. The ambient PPFD was measured with a micro-quantum sensor connected to a Licor 1400 data logger. In the present study, 3 min of 635 nm red actinic LED light corresponding to the natural PPFD at a given solar time was used for measurements of chlorophyll fluorescence and P700 redox state. The time lag between measured PPFD and the onset of the measurements of the fluorescence and P700 was 3 min. During the time lag, the measured leaves were incubated in darkness, which favors the later measurement of the maximum quantum yield of P700 (*P_m_*). After measuring light-adapted chlorophyll fluorescence and P700 redox state, the leaves were incubated in darkness for 20 min and then the maximum quantum yield of PSII (*F_v_*/*F_m_*) and *P_m_* were measured. There were no significant differences between the values of *P_m_* measured after 3 or 20 min incubation in darkness in the two species. As a result, we used the value of *P_m_* measured after 3 min incubation in darkness in our present study.

The fluorescence parameters were calculated as follows: *F_v_*/*F_m_* = (*F_m_* -*F_o_*)/*F_m_*, *F_o_′* = *F_o_*/(*F_v_*/*F_m_* + *F_o_*/*F_m_′*) (Oxborough and Baker, 1997), *F_v_′/F_m_′* = (*F_m_′*- *F_o_′*)/*F_m_′*, qP = (*F_m_′*-*F_s_*)/(*F_m_′*-*F_o_′* ), Y(II) = (*F_m_′*-*F_s_*)/*F_m_′* (Genty et al., 1989), Y(NO) = *F_s_*/*F_m_*, Y(NPQ) = *F_s_*/*F_m_′*-*F_s_*/*F_m_* (Hendrickson et al., 2004b; Kramer et al., 2004), where *F_o_* and *F_o_′* are the minimum fluorescence values in the dark-adapted and light-adapted states, respectively. Notably, the value of *F_o_′* was calculated according to the above equation. *F_m_* and *F_m_*′ are the maximum fluorescence values upon illumination of pulse (300 ms) of saturating light (10000 μmol m^-2^ s^-1^) in the dark-adapted and light-adapted state, respectively. *F_s_* is the steady state chlorophyll fluorescence value in a light-acclimated state. Y(II) is the effective quantum yield of PSII. Y(NO) is the quantum yield of non-regulated energy dissipation. Y(NPQ) is the fraction of energy dissipated in form of heat via the regulated NPQ mechanism.

The PSI parameters were measured with a dual wavelength (830/875 nm) unit, following the method of Klüghammer and Schreiber (1994, 2008). Saturation pulses (10000 μmol photons m^-2^ s^-1^), were applied for assessment of P700 parameters as well (Klüghammer and Schreiber, 2008). The P700^+^ signals (*P*) may vary between a minimal (P700 fully reduced) and a maximal level (P700 fully oxidized). The maximum level, which in analogy to *F_m_* is called *P_m_*, was determined with application of a saturation pulse during illumination with far-red light. At a defined optical property, the amplitude of *P_m_* depends on the maximum amount of photo-oxidizable P700, which is a good parameter for representing PSI activity (Huang et al., 2010a,b, 2013; Suorsa et al., 2012; Kono et al., 2014; Tikkanen et al., 2014). *P_m_′* was also defined in analogy to the fluorescence parameter *F_m_′*. *P_m_′* was determined similarly to *P_m_*, but with background actinic light instead of far-red illumination. The photochemical quantum yield of PSI, Y(I), is defined by the fraction of overall P700 that in a given state is reduced and not limited by the acceptor side. It is calculated as Y(I) = (*P_m_′*- *P*)/*P_m_*. Y(ND) = *P*/*P_m_*, represents the fraction of P700 that is already oxidized in a given state. Y(NA) = (*P_m_* -*P_m_′*)/*P_m_*, thus represents the fraction of P700 that cannot be oxidized by a saturation pulse to the overall P700.

Photosynthetic electron flow through PSI and PSII was calculated as follows: ETRI = PPFD × Y(I) × 0.85 × αI, ETRII = PPFD × Y(II) × 0.85 × αII. 0.85 is assumed to be the leaf absorbance. αI and αII represent the fractions of the absorbed light distributed to PSI and PSII, respectively. In our present study, αI and αII are calculated using values of Y(I) and Y(II) under 18 μmol photons m^-2^ s^-1^ according to the method of Huang et al. (2012) and Zivcak et al. (2013).

### Statistical Analysis

The results were displayed as mean values of four to six independent experiments. The data were subjected to analysis of variance (ANOVA) using the SPSS 16.0 statistical software. Tukey’s multiple comparison test was used at α = 0.05 significance level to determine whether significant differences exist among different treatments.

## Results

### Light Response Changes in Energy Quenching in PSII and P700 Redox State

**FIGURE 2:**
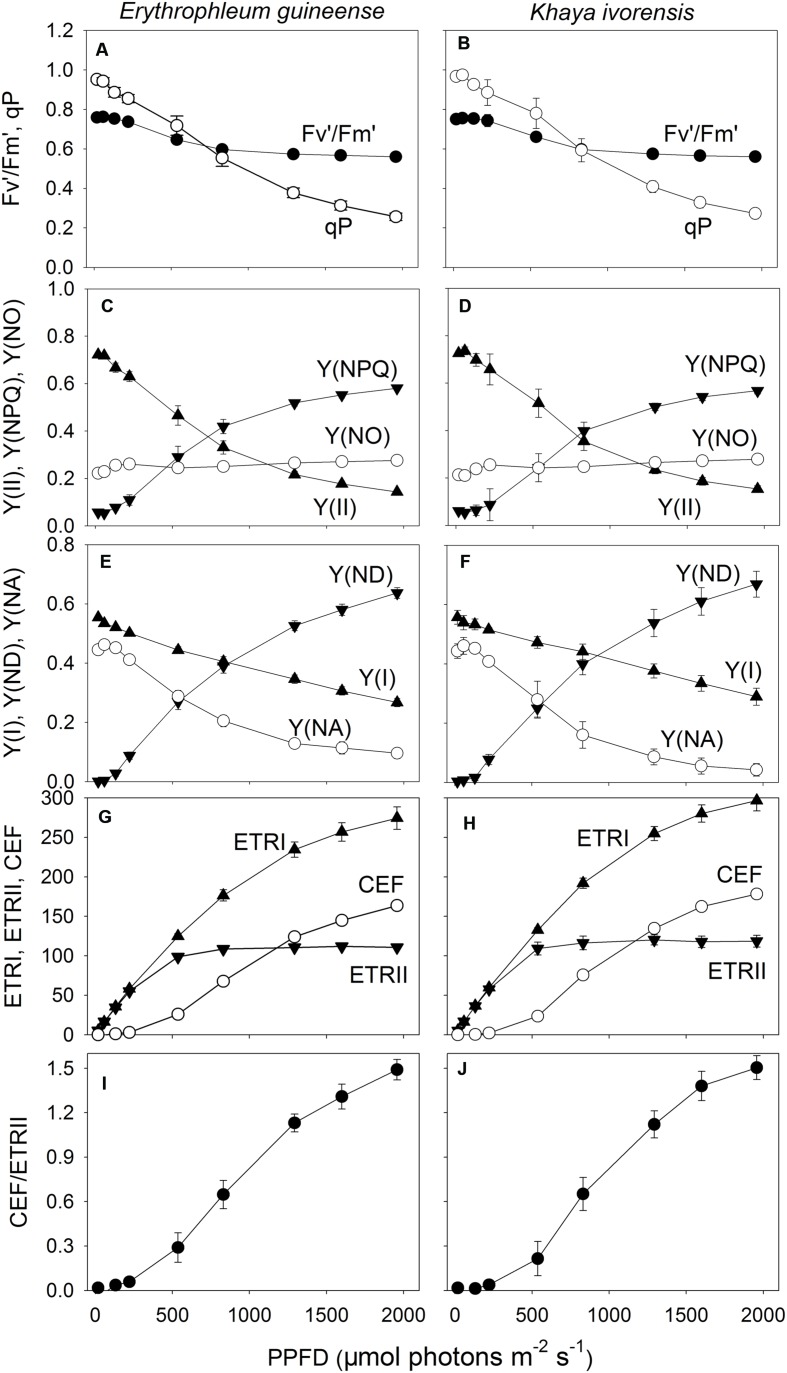
**Light response changes (A–J) in *F_v_′/F_m_′*, qP, Y(II), Y(NPQ), Y(NO), Y(I), Y(ND), Y(NA), ETRI, ETRII, CEF, and CEF/ETRII ratio for leaves of *E. guineense* and *K. ivorensis* measured at 25°C without photoinhibition**.*F_v_′/F_m_′*, maximum quantum yield of PSII under light; qP, coefficient of photochemical quenching; Y(II), effective quantum yield of PSII; Y(NPQ), fraction of energy dissipated in form of heat via the regulated non-photochemical quenching mechanism; Y(NO), fraction of energy that is passively dissipated in form of heat and fluorescence; Y(I), effective quantum yield of PSII; Y(ND), fraction of overall P700 that is oxidized in a given state; Y(NA), fraction of overall P700 that cannot be oxidized in a given state; ETRI, photosynthetic electron flow through PSI; ETRII, photosynthetic electron flow through PSII; CEF, cyclic electron flow. The mean ± SE was calculated from six plants.

With increasing light intensity, the decrease in qP was larger than that of *F_v_′/F_m_′*. As a result, the effective quantum yield of PSII [Y(II)] gradually decreased with increasing light intensity, mainly due to the decrease in qP (**Figures 2A–D**). Meanwhile, the fraction of energy dissipated as heat via the regulated [Y(NPQ)] strongly increased (**Figures 2C,D**). The quantum yield of non-regulated energy dissipation in PSII [Y(NO)] was maintained stable near the baseline of 0.2 in the two species (**Figures 2C,D**). The value of quantum yield of PSI [Y(I)] gradually decreased with increasing light intensity in the two species (**Figures 2E,F**). Meanwhile, the faction of P700 that is already oxidized in a given state [Y(ND)] largely increased with an increase in light intensity. The fraction of overall P700 that cannot be oxidized in a given state [Y(NA)] was maintained at a low level of approximately 0.1 under high light in the two species (**Figures 2E,F**).

At light intensities below 221 μmol photons m^-2^ s^-1^, the value of ETRI approximately equaled ETRII, resulting in little activation of CEF (**Figures 2G,H**). Values for ETRII reached the maximum at a light of 830 μmol photons m^-2^ s^-1^, but ETRI gradually increased with an increase in light intensity in both species (**Figures 2G,H**). Accordingly, CEF was activated at light intensities above 536 μmol photons m^-2^ s^-1^ and increased gradient with increasing light intensity. The CEF/ETRII ratio showed similar trend as CEF (**Figures 2I,J**). Combining with the light response changes in Y(NPQ), Y(ND), and Y(NA), the large difference in CEF/ETRII ratio between low light and high light indicated the stimulation of CEF around PSI under high light in both species.

### Diurnal Change in Energy Distribution in PSII and P700 Redox State

**FIGURE 3:**
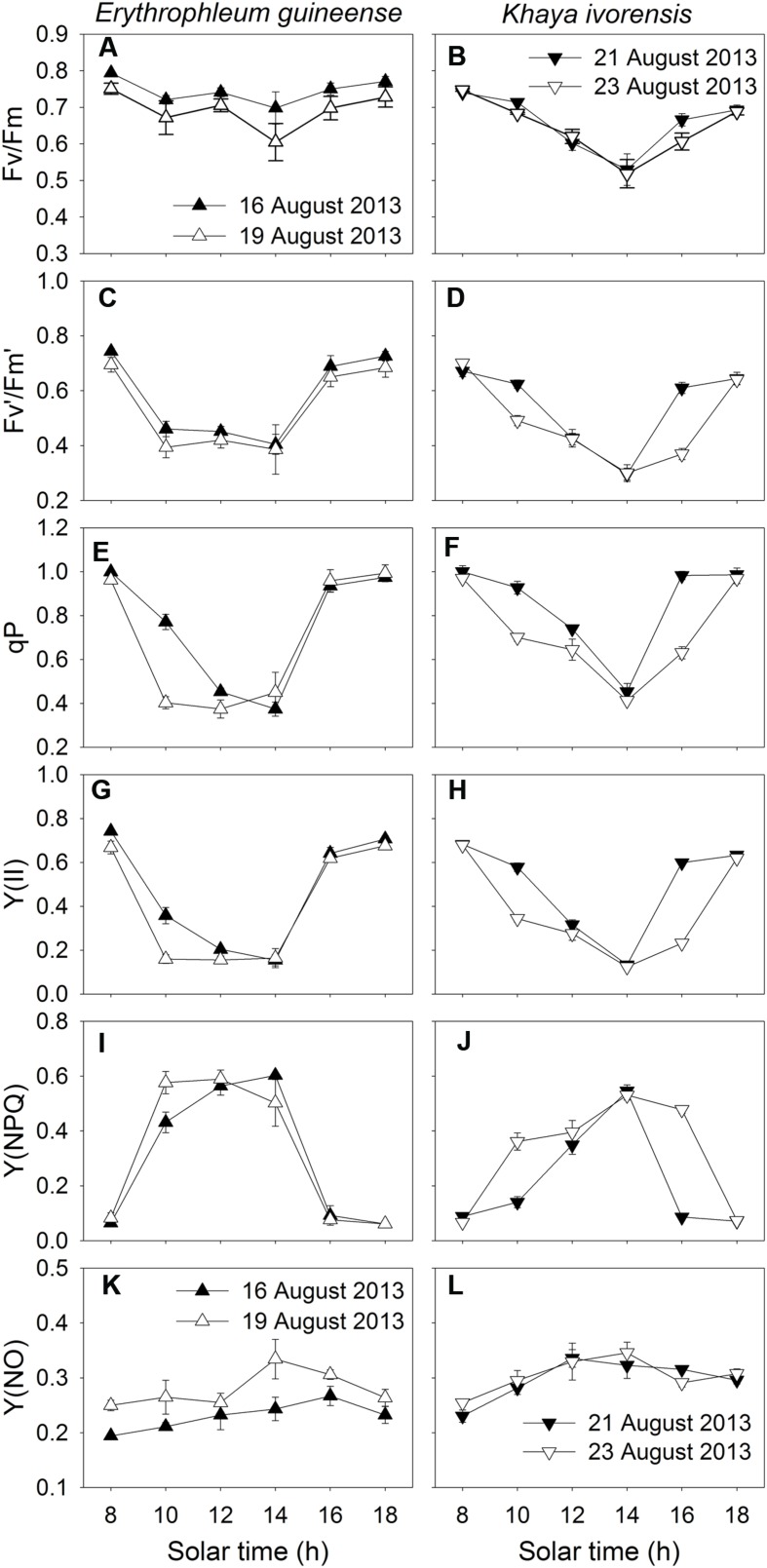
**Diurnal Changes (A–L) in *F_v_/F_m_*, *F_v_′/F_m_′*, qP, Y(II), Y(NPQ), and Y(NO) for leaves of *Erythrophleum guineense* and *Khaya ivorensis* in summer**. The mean ± SE were calculated from at least four plants.

During clear summer days, the maximum quantum yield of PSII (*F_v_/F_m_*) decreased significantly at noon and recovered fast in the afternoon in both species (**Figures 3A,B**), indicating the high-light-induced PSII photoinhibition. At noon, both *F_v_′/F_m_′* and qP significantly decreased, leading to a decrease in Y(II) (**Figures 3C–H**). In the afternoon, when the light intensity was low, Y(II) fast recovered due to an increase in both *F_v_′/F_m_′* and qP (**Figures 3C–H**). The value of Y(NPQ) was maintained at a low level in the early morning and the late afternoon, but largely increased at noon to dissipate excess absorbed light energy in the two species (**Figures 3I,J**). The value of Y(NO) increased at noon and decreased in the afternoon, indicating a detrimental effect of excess light energy, at noon, on PSII (**Figures 3K,L**).

**FIGURE 4:**
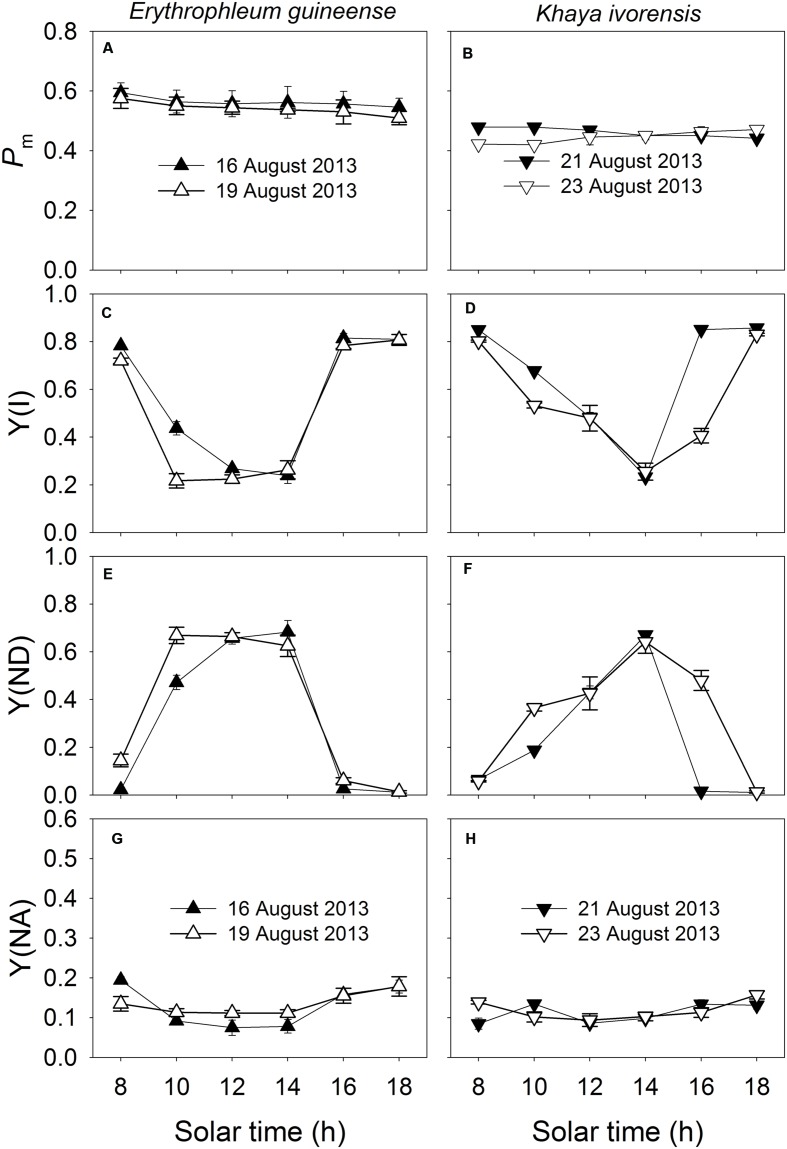
**Diurnal Changes (A–H) in *P_m_*, Y(I), Y(ND), and Y(NA) for leaves of *E. guineense* and *K. ivorensis* in summer**. The mean ± SE were calculated from at least four plants.

The *P_m_* was maintained stable during the daytime (**Figures 4A,B**), indicating the maintenance of the stable amount of P700 active reactions centers and thus stable activity of PSI during the daytime. Since PSI photoinhibition can be affected by the redox state of P700, values for Y(I), Y(ND), and Y(NA) were measured to examine the diurnal change in P700 redox state. The value of Y(I) significantly decreased at noon and recovered fast in the afternoon (**Figures 4C,D**). Under high light at noon, Y(ND) was maintained at high levels at noon, and Y(NA) was maintained at low level of approximately 0.1 (**Figures 4E–H**), indicating the over-reduction of PSI acceptor side was prevented under high light. At low light intensities in the afternoon, both species showed low levels of Y(ND) (**Figures 4E,F**).

**FIGURE 5:**
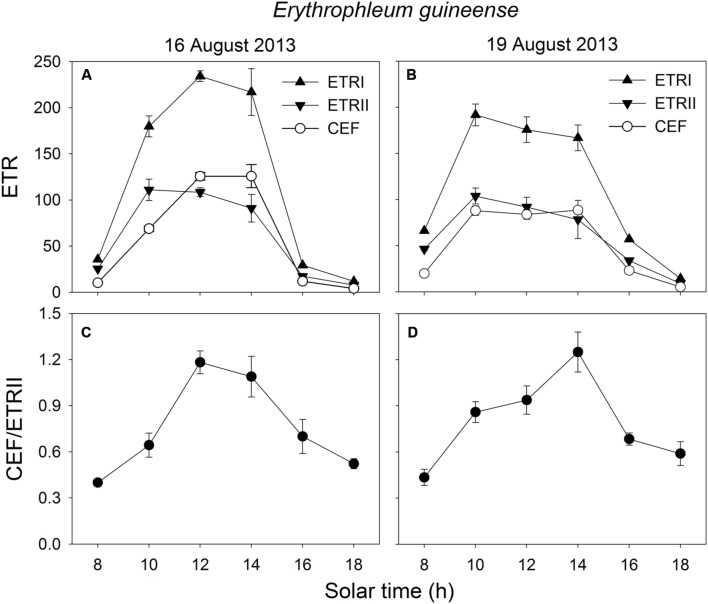
**Diurnal Changes (A–D) in ETRI, ETRII, CEF, and CEF/ETRII ratio for leaves of *E. guineense* in summer**. The mean ± SE were calculated from at least four plants.

**FIGURE 6:**
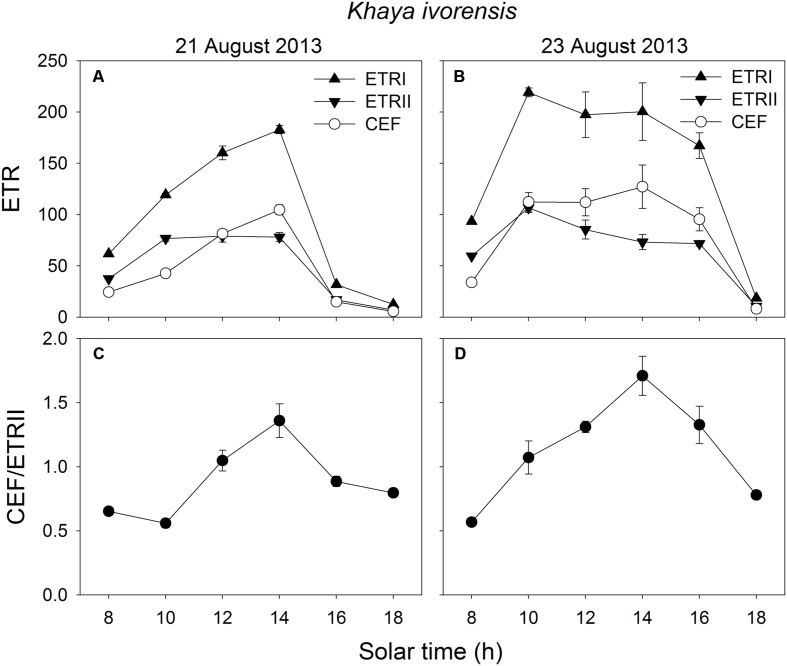
**Diurnal Changes (A–D) in ETRI, ETRII, CEF, and CEF/ETRII ratio for leaves of *K. ivorensis* in summer**. The mean ± SE were calculated from at least four plants.

In the early morning, the values of ETRI, ETRII, and CEF were low in both species. The value of CEF/ETRII ratio at 8:00 was approximately 0.4 in *E. guineense* and 0.6 in *K. ivorensis* (**Figures 5** and **6**). ETRI and ETRII reached their maximum values at around 12:00 in both species (**Figures 5** and **6**). The value of CEF reaches its maximum value at approximately 14:00 (**Figures 5** and **6**). As a result, both species showed the maximum value of CEF/ETRII ratio approximately at 14:00 (**Figures 5** and **6**). In *E. guineense*, the maximum CEF/ETRII ratio on 16 August and 19 August were 1.18 and 1.25, respectively (**Figure 5**). In *K. ivorensis*, the maximum CEF/ETRII ratio on 21 August and 23 August were 1.36 and 1.71, respectively (**Figure 6**). In the afternoon, both species showed a significant stimulation in the CEF at low light, and the value of CEF/ETRII ratio changed from 0.5 to 0.8 in the two species (**Figures 5** and **6**). The value of CEF/ETRII ratio at 18:00 was significantly higher than that under a PPFD of 58 μmol photons m^-2^ s^-1^ measured without PSII photoinhibition in both species (**Figures 2** and **6**). These results indicated that CEF was activated not only under high light at noon, but also under low light in the late afternoon.

## Discussion

### Estimation of the Rate of CEF

Cyclic electron flow was discovered 50 years ago by Arnon (1959) and Tagawa et al. (1963). They have used *in vitro* system to study CEF, mainly by measuring the O/P stoichiometry on isolated thylakoids and chloroplasts. However, it is complicated to distinguish between the main cyclic route and pseudocyclic (water-water cycle) till date. To understand the physiological function of CEF during a clear day, the activity of CEF should be evaluated. Three methods for the determination of CEF activity have been proposed and all depend on the exact determination of the P700 turnover rate (Miyake, 2010). The first was developed by Klüghammer and Schreiber (1994) group. The second was developed by Johnson (2003) and Nandha et al. (2007) group and the third was developed by Joliot and Joliot (2006) and Baker et al. (2007) group. The three methods reported an accurate result showing that CEF operates at a higher activity during the induction phase of photosynthesis (Joliot and Joliot, 2002; Maniko et al., 2002). Until now, Schreiber’s method has been widely used to study or investigate CEF (Suorsa et al., 2012; Kono et al., 2014; Tikkanen et al., 2014, 2015; Kou et al., 2015). In our present study, we used Schreiber’s method to determine CEF activity. Light response changes in Y(ND), Y(NA) and CEF in both studied species (**Figure 2**) were similar to those obtained in leaves of *Arabidopsis* wild-type (Kono et al., 2014; Kou et al., 2015; Tikkanen et al., 2015). This supports our findings and indicates the reliability and accuracy of this method.

### Stimulation of CEF at Noon

We found that CEF was strongly stimulated at noon in the two species (**Figures 5** and **6**). At noon in a clear day, the increase in air temperature can increase both leaf temperature and the leaf-to-air vapor pressure deficit, which leads to partial stomatal closure and depression of photosynthesis (Zhang et al., 2009). Several studies reported that CEF activity could be enhanced by heat stress in potato and spinach (Havaux, 1996; Bukhov et al., 1999; Kou et al., 2013). However, the decrease of CEF due to heat stress may occur in some heat-sensitive plant genotypes (Essemine et al., 2011; Brestic et al., 2016). Plants have the ability to quench excess light energy in PSII through NPQ, which is based on build-up of ΔpH across thylakoid membranes (Munekage et al., 2002, 2004; Takahashi et al., 2009). CEF is necessary for the normal activation of NPQ in *Arabidopsis* illuminated under high light (Munekage et al., 2002, 2004; Nandha et al., 2007; Takahashi et al., 2009). In our present study, CEF was significantly stimulated at noon (**Figures 5** and **6**), which was accompanied with activation of Y(NPQ) (**Figures 3I,J**). These results suggested that CEF plays an important role in the activation of NPQ at noon, which may alleviate the production of ROS. It has been reported that ROS inhibit the repair of photoinhibited PSII (Nishiyama et al., 2001, 2004; Takahashi et al., 2009). Thus, it is very likely that the highly activation of CEF, at noon, favored the repair of photodamaged PSII.

Furthermore, some studies proposed that photodamage of PSII primarily occurred at the oxygen-evolving complex that is located on the luminal side of thylakoid membrane (Hakala et al., 2005; Ohnishi et al., 2005). Previous study suggested that a high concentration of Ca^2+^ in the lumen of thylakoids could stabilize the oxygen-evolving complex against photodamage (Krieger and Weis, 1993). Since acidification of the lumen could drive a Ca^2+^/H^+^ antiport to sequester Ca^2+^ in the lumen, up to about 4 mM in the lumen from an external concentration of 15 μM (Ettinger et al., 1999), impairment of the generation of ΔpH across the thylakoid membrane caused acceleration of the photodamage to PSII (Takahashi et al., 2009). Furthermore, the inhibition of CEF-dependent formation of ΔpH could induce severe photodamage to PSII under high light (Takahashi et al., 2009; Tikkanen et al., 2014). Our recent study indicated that CEF played a significant role in the photoprotection for the oxygen-evolving complex (Huang et al., 2016). The present results indicate a slightly or moderately photoinhibition of PSII during clear days. Therefore, the strong stimulation of CEF at noon likely protects PSII against photoinhibition through stabilizing the oxygen-evolving complex.

Our results indicated that the strong stimulation of CEF at noon prevents the PSI photoinhibition. Generally, PSI is insusceptible to high light stress in wild-type plants, which is partly explained by the high proportion of P700 accumulated in the oxidized state (Barth et al., 2001). Munekage et al. (2002, 2004) have reported that PGR5-dependent CEF is essential for photoprotection of PSI in *Arabidopsis* as evidenced by the small fraction of oxidized P700 in the *pgr5* plants. Furthermore, a recent study indicated that PGR5-dependent CEF is necessary for PSI activity under fluctuating light conditions (Suorsa et al., 2012). PGR5-dependent CEF is responsible for photosynthetic control of electron transfer, which protects PSI from over-reduction and oxidative damage (Suorsa et al., 2012; Tikkanen et al., 2014). Since PSI photoinhibition is induced by the over accumulation of hydroxyl radicals which are generated between reduced PSI reaction centers and hydrogen peroxide (Sonoike, 1996a,b, 2006, 2011), the over-reduction of PSI acceptor side is a prerequisite for photoinhibition of PSI. In the present study, the stimulation of CEF increased the oxidation ratio of P700 and decreased the fraction of P700 that cannot be oxidized (**Figure 4**), indicating that the stimulation of CEF at noon prevents the over-reduction of PSI acceptor side. It was reported that high-light stress could decrease the fraction of photo-oxidized P700 in some plant grown in field (Endo et al., 2005). However, our results indicate that a large fraction of P700 was oxidized under high light at noon in the two species. This could occur by the diversion of electrons from reduced ferredoxin into CEF.

Furthermore, the over-accumulation of reducing power NADPH, resulting in generation of hydroxyl radicals in PSI reaction centers (Murata et al., 2007), is documented as a necessary mechanism for the photoinhibition of PSI (Wang et al., 2006; Shikanai, 2007). The inhibition of CO_2_ fixation at noon could induce the over-accumulation of NADPH so that could increase the risk of PSI photoinhibition. CEF could consume excess NADPH through the NADPH dehydrogenase-dependent pathway (Shikanai, 2007). Chloroplastic NAD(P)H dehydrogenase in tobacco leaves functions in alleviation of oxidative damage caused by high temperature stress (Wang et al., 2006). The strong stimulation of CEF at noon suggested that it may protect PSI from photoinhibition through alleviating the over-accumulation of NADPH.

### Stimulation of CEF in the Afternoon

In addition to photoprotection, another important role of CEF is to help extra ATP synthesis, which is necessary for optimal photosynthesis and PSII repair. Our results indicated that CEF was significantly stimulated at low light in the afternoon (**Figures 5** and **6**), which was accompanied with the fast repair of photodamaged PSII (**Figure 3**). The main feature of the repair process is the replacement of the D1 protein in the photodamaged PSII complexes by newly synthesized D1 and reassembly of active PSII (Guenther and Melis, 1990; Aro et al., 1993; Kettunen et al., 1997; Allakhverdiev et al., 2005). The fast repair of PSII photoinhibition is dependent on ATP synthesis (Allakhverdiev et al., 2005), which is in turn dependent on the formation of ΔpH across thylakoid membranes. Our previous study suggested that CEF was stimulated under a low light to help the recovery of chilling-induced photoinhibition of PSII (Huang et al., 2010b). CEF-mutants of *A. thaliana*, *ccr6* and *pgr5*, showed lower rate of CO_2_ assimilation under low light than wild type, suggesting that CEF activity could be important for ATP synthesis at low light (Yamori et al., 2011; Nishikawa et al., 2012). In the present studied two species, CEF was activated under low light in the late afternoon (**Figures 5** and **6**). Meanwhile, the values of Y(NPQ) and Y(ND) were maintained at low levels (**Figures 5** and **6**). These results indicated that, at low light in the afternoon, CEF-dependent generation of ΔpH did not cause luminal acidification. As a result, in the afternoon, the CEF-dependent generation of ΔpH probably contributed to ATP synthesis. These results suggest that the stimulation of CEF in the afternoon mainly helps the repair of PSII photoinhibition rather than contributes to photoprotection.

### The Physiological Significance of Stability of PSI Activity in the Daytime

The present study showed that high light caused PSII photoinhibition in sun leaves at noon whereas the PSI activity remained very stable (**Figures 3** and **4**). A possible reason for the preference of maintaining stable PSI activity is that the repair of PSII activity is fast but the repair of PSI activity is relatively slow (Zhang and Scheller, 2004). Furthermore, the fast recovery of photodamaged PSII was dependent on a moderate PSI activity. Moderate PSI photoinhibition slowed the rate of PSII recovery (Kudoh and Sonoike, 2002), and severe PSI photoinhibition resulted in failure of recovery of both PSI and PSII from photoinhibition (Huang et al., 2010a). Therefore, the stability of PSI activity during the daytime contributed to the photoprotection and recovery of PSII activity. Additionally, the decrease of CEF-dependent formation of ΔpH due to damage of PSI led to a substantial decrease of photosynthetic CO_2_ assimilation, especially at low light (Zivcak et al., 2015). Because the operation of CEF involves the assembly of super complex including PSI complex (Peng and Shikanai, 2011), we speculate that the main role of stability of PSI activity in the daytime is to guarantee the activation of CEF.

In summary, our results indicate that CEF was not only activated under high light at noon but as well at low light in the afternoon. The stimulation of CEF at noon has mainly two functions: one is alleviating PSII photoinhibition, and the other is preventing PSI photoinhibition. It is presumably that the stimulation of CEF during the afternoon helps mainly for the fast repair of PSII photoinhibition via ATP synthesis. The stability of PSI activity in the daytime guaranteed the stimulation of CEF and in turn favored photoprotection and repair of PSII photoinhibition.

## Author Contributions

WH and S-BZ conceived and designed research. WH and Y-JY conducted experiments. WH, Y-JY, and S-BZ analyzed data. WH, Y-JY, HH, K-FC, and S-BZ wrote the manuscript.

## Conflict of Interest Statement

The authors declare that the research was conducted in the absence of any commercial or financial relationships that could be construed as a potential conflict of interest.

## Conflict of Interest Statement

The authors declare that the research was conducted in the absence of any commercial or financial relationships that could be construed as a potential conflict of interest.
